# The subxiphoid view cannot replace the apical view for transthoracic echocardiographic assessment of hemodynamic status

**DOI:** 10.1186/cc12869

**Published:** 2013-09-03

**Authors:** Julien Maizel, Ahmed Salhi, Christophe Tribouilloy, Ziad A Massy, Gabriel Choukroun, Michel Slama

**Affiliations:** 1 Medical Intensive Care Unit, Department of Nephrology, Amiens University Medical Center, Amiens, France and INSERM U-1088, Jules Verne University of Picardie, Amiens, France; 2 Department of Cardiology, Amiens University Medical Center, Amiens, France and INSERM U-1088, Jules Verne University of Picardie, Amiens, France

## Abstract

**Introduction:**

This prospective study aimed to assess whether use of the subxiphoid acoustic window in transthoracic echocardiography (TTE) can be an accurate alternative in the absence of an apical view to assess hemodynamic parameters.

**Methods:**

This prospective study took place in a teaching hospital medical ICU. Over a 4-month period, TTE was performed in patients admitted for more than 24 hours. Two operators rated the quality of parasternal, apical, and subxiphoid acoustic windows as Excellent, Good, Acceptable, Poor, or No image. In the subpopulation presenting adequate (rated as acceptable or higher) apical and subxiphoid views, we compared the left ventricular ejection fraction (LVEF), the ratio between right and left ventricular end-diastolic areas (RVEDA/LVEDA), the ratio between early and late mitral inflow on pulsed Doppler (E/A ratio), the aortic velocity time integral (Ao VTI), and the ratio between early mitral inflow and displacement of the mitral annulus on tissue Doppler imaging (E/Ea ratio).

**Results:**

An adequate apical view was obtained in 80%, and an adequate subxiphoid view was obtained in 63% of the 107 patients included. Only 5% of patients presented an adequate subxiphoid view without an adequate apical view. In the subpopulation of patients with adequate apical and subxiphoid windows (*n *= 65), LVEF, E/A, and RVEDA/LVEDA were comparable on both views, and were strongly correlated (*r *> 0.80) with acceptable biases and precision. However, the Ao VTI and the E/Ea ratio were lower on the subxiphoid view than on the apical view (18 ± 5 versus 16 ± 5 cm and 9.6 ± 4.6 versus 7.6 ± 4 cm, respectively, *P *= 0.001 for both).

**Conclusions:**

An adequate TTE subxiphoid window was obtained in fewer than two thirds of ICU patients. In addition to the classic indication for the subxiphoid window to study the vena cava and pericardium, this view can be used to study right and left ventricular morphology and function, but does not provide accurate hemodynamic Doppler information. ICU echocardiographers should therefore record both apical and subxiphoid views to assess comprehensively the cardiac function and hemodynamic status.

## Introduction

Transthoracic echocardiography (TTE) has become the leading noninvasive examination to assess the hemodynamic status of patients hospitalized in intensive care units (ICUs) [1-3]. It allows the physician to study noninvasively the contractility, relaxation, and fluid responsiveness of the two ventricles [4]. All parameters must be recorded in one of the following three windows: parasternal, apical, or subxiphoid. As recommended in guidelines, the appropriate window corresponds to the Doppler mode with the best alignment obtained between the ultrasound beam and the direction of blood flow [5]. For that reason, most parameters recorded for hemodynamic assessment classically require an apical window: left ventricular ejection fraction (LVEF) [6,7], ratio between early and late mitral inflow (E/A) [8], ratio between early mitral inflow and displacement of the mitral annulus on tissue Doppler imaging (E/Ea) [8], velocity time integral of aortic blood flow (Ao VTI) [9], and the ratio between right (RVEDA) and left (LVEDA) ventricular end-diastolic area [7]. However, many factors in the ICU can interfere with apical image quality. More specifically, in this setting, the lung interposition between the probe and the heart, the presence of mechanical ventilation, the inability to position the patient in left-sided decubitus, tachypnea, and the presence of bandages or tubes on the chest can alter the quality of the images obtained on apical and parasternal views.

To complete the examination in this setting, the echocardiographer may perform transesophageal echocardiography. However, transesophageal echocardiography is more invasive and can be difficult to perform, especially in nonintubated ICU patients. In this setting, use of the transthoracic subxiphoid view can be an attractive alternative to transesophageal echocardiography for less-experienced operators who may be reluctant to perform an invasive examination. The subcostal view is classically used to study respiratory variations of the inferior vena cava and the presence of pericardial effusion. This view can provide additional hemodynamic parameters, such as LVEF, mitral or aortic flows, and RV/LV areas. Although the subxiphoid view does not provide the same alignment as the apical view, it is not known to what degree this different alignment affects the results of the hemodynamic examination.

To address this issue, we tried to determine the accuracy of the subxiphoid window to record various echocardiographic parameters usually recorded on the apical view for hemodynamic assessment.

## Materials and methods

### Patients

In accordance with French legislation, the local institutional review board (CPP Nord-Ouest II, Amiens University Hospital, France) approved the study protocol, and all patients or their relatives gave their informed consent. This single-center prospective study was performed in an eight-bed teaching hospital medical ICU (Amiens, France). Over a 4-month period, all patients admitted to the ICU were included in the study, except when one of the two experienced echocardiographers (JM and AS) was absent and except for patients admitted to the unit for less than 24 hours. Patient characteristics were recorded.

### Measurements and analyses

Transthoracic echocardiography was performed during the first 2 days after admission in all patients by one of the two operators (JM or AS) by using a Vivid i (General Electrics Healthcare Fairfield, CT, USA) equipped with a 3-MHz probe. Parasternal (long and short axis), apical four-chamber, and subxiphoid four-chamber views were obtained successively in 2D mode, and a cine-loop was stored for offline analysis. In the apical and subxiphoid windows, pulsed Doppler mitral inflow was recorded with the sample cursor placed at the mitral valve tips; early diastolic velocity (Ea) was determined from Doppler tissue imaging with the sample cursor placed in the lateral mitral annulus, and aortic blood flow was measured with the sample volume placed at the aortic annulus. The size of the sample volume was not modified between apical and subxiphoid views.

The two operators then blindly reviewed the quality of the 2D images recorded with these windows offline on a computer (General Electric Healthcare, Fairfield, CT, USA), according to the following scoring system: Excellent (visualization of the four chambers with complete endocardial visualization of both ventricles); Good (visualization of the two ventricles with complete endocardial visualization of both ventricles); Acceptable (visualization of both ventricles, but short segments of endocardium are not correctly visualized); Poor (the endocardium cannot be visualized in most portions of the ventricles); and No image (no visualization of the ventricles). An adequate acoustic window was defined by an acceptable, good, or excellent score. Each physician separately scored the video recording of the three windows (parasternal, apical, and subxiphoid) and compared their results. When both scores were identical, the final score was accepted. When the two scores were different, the physicians jointly visualized the video, discussed their points of view, and reached a consensus to establish a final score.

In the subpopulation of patients with adequate apical and subxiphoid windows (scored excellent, good, or acceptable), LVEF (according to the Monoplane Simpson method) and RVEDA/LVEDA ratio were measured on both the apical and subxiphoid 2D cine-loops. The mitral peak E velocity (E), peak A velocity (A), E/A ratio, Ao VTI, and E/Ea ratio were measured on Doppler images in the apical and subxiphoid windows. All data were the average of three end-expiratory measurements (excluding outliers). In the presence of an outlier, an additional assessment was recorded. In the subxiphoid window, LVEF and RVEDA/LVEDA were recorded on a longitudinal four-chamber view visualizing both right and left ventricles in their largest dimensions. On a modified four-chamber view, the operator tried to obtain the best alignment between flow and the Doppler axis by obtaining the highest maximum Doppler velocity to record mitral and aortic flows on the subxiphoid view.

### Statistics

All variables are expressed as mean ± 1 SD or proportions. The normal distribution was tested with a Kolmogorov-Smirnov test. Quantitative variables were compared by using a Student *t *test, and proportions were compared with a χ^2 ^test.

Pearson correlation coefficient and Bland-Altman analysis were used to assess the adequacy of the various parameters obtained in the subxiphoid and apical windows. This analysis was performed in the subpopulation of patients in whom apical and subxiphoid windows were scored as excellent, good, or acceptable (*n *= 65). The bias and limit of agreement (LOA) around the bias were given by the Bland-Altman representation. However, no consensus has been reached concerning the acceptable LOA to confirm validation of the subcostal view. To define an acceptable LOA, Cecconi *et al*. [10] proposed assessing the precision of the reference technique (in this case, the apical view) and the tested technique (the subxiphoid view). We prospectively defined the validation criteria for the various parameters studied on the subxiphoid view: to be acceptable, the parameter on the subxiphoid view had to be accurate (bias <10%) and precise (a lower percentage precision value than on the apical view).

The inter- and intraobserver reproducibilities were determined for each parameter (LVEF, E/A, E/Ea, RVEDA/LVEDA, and Ao VTI) in both the apical and subxiphoid windows. All intraobserver variabilities were <5%, and interobserver variabilities were <9%.

Statistical analysis was performed by using MedCalc version 12.0.4.0 (MedCalc Software, Mariakerke, Belgium). The limit of significance was set at ≤ 0.05.

## Results

### Demographic data

Patient characteristics are presented in Table 1. Supraventricular arrhythmia (atrial fibrillation and atrial flutter) was reported in 23 (21%) patients. The admission diagnoses were shock in 35%, respiratory distress in 23%, acute renal failure in 10%, cardiac arrest in 5%, severe sepsis in 4%, deliberate drug overdose in 5%, scheduled postoperative surveillance in 5%, and other (seizures, pancreatitis, ketoacidosis) in 13%.

**Table 1:** Patient characteristics of the overall population

	Overall population *N *= 107
**Age (years)**	63 ± 16
Men, *n *(%)	59 (55)
SAPS II	49 ± 20
BMI, kg/m^2^	28 ± 8
COPD, n (%)	26 (24)
Mechanical ventilation, *n *(%) VC PSV PEEP, cm H_2_O	48 (45) 40 (37) 8 (7) 3.4 ± 1.7
MAP, mm Hg	79 ± 17
HR, bpm	97 ± 23
Arrhythmia, *n *(%)	23 (21)
Catecholamines, *n *(%)	36 (34)
Tubes, bandages, *n *(%) Chest Abdomen	10 (9) 6 (6) 4 (4)

BMI, body mass index; COPD, chronic obstructive pulmonary disease; HR, heart rate; MAP, mean arterial pressure; PEEP, positive end-expiratory pressure; PSV, pressure-support ventilation; SAPSII, simplified acute physiology score II; VC, volume controlled.

### Measurements

The mean number of adequate acoustic windows per patient was 2.2 ± 0.9. An adequate parasternal view was obtained in 69% of patients; an adequate apical view was obtained in 86% of patients; and an adequate subxiphoid view was obtained in 66% of patients. The proportion of adequate views was significantly higher for the apical window than for the subxiphoid window for the overall population and the subpopulation of mechanically ventilated patients and was close to significant in COPD patients (Table 2). Three adequate views were obtained in 45% of patients; two adequate views were obtained in 37%; only one adequate view was obtained in 10%; and no adequate window was obtained in 8% of patients. An adequate subxiphoid view but no adequate apical view was obtained in only 5% of patients. The proportion of adequate subxiphoid windows was not affected by mechanical ventilation (67% versus 66%; *P *= 0.9 with or without mechanical ventilation, respectively), the mode of ventilation (67% versus 62%; *P *= 0.9 in VC or PSV mode, respectively), the level of PEEP (67% versus 73%; *P *= 0.9, PEEP <5 and PEEP ≥5 cm H_2_O, respectively), or a history of COPD (58% versus 69%; *P *= 0.4 COPD versus absence of COPD, respectively).

**Table 2:** Quality of the various windows obtained and proportion of each parameter recorded in apical and subxiphoid windows in the overall population (*n *= 107)

	Apical	Subxiphoid	*P*
Number of patients with adequate apical or subxiphoid acoustic window, *n *(%) Overall population (*n *= 107) Mechanically ventilated patients (*n *= 48) COPD population (*n *= 26)	92 (86) 38 (78) 21 (81)	71 (66) 32 (67) 15 (58)	0.009 0.002 0.1
Quality of the window A Excellent B Good C Acceptable D Poor E No image	27 (25) 36 (34) 29 (27) 11 (10) 4 (4)	19 (18) 36 (34) 16 (15) 21 (20) 15 (14)	0.7 0.3 0.9 0.06 0.2
LVEF, *n *(%)	80 (75)	67 (63)	0.001
E/A, *n *(%)	71 (66)	46 (43)	0.001
E/Ea, *n *(%)	85 (79)	66 (62)	0.001
Ao VTI, *n *(%)	83 (78)	66 (62)	0.001
RVEDA/LVEDA, *n *(%)	79 (74)	67 (63)	0.001

Ao VTI, velocity time integral of aortic blood flow; E/A, ratio between early and late mitral inflow; E/Ea, ratio between early mitral inflow and displacement of the mitral annulus on tissue Doppler imaging; LVEF, left ventricular ejection fraction; RVEDA/LVEDA, ratio between right and left ventricular end-diastolic area.

A higher mean number of the five parameters (LVEF, Ao VTI, E/A, E/Ea, and RVEDA/LVEDA) was obtained on the apical view than on the subxiphoid view (3.7 ± 1.9 and 2.9 ± 2.2, respectively; *P *= 0.001). When considered separately, each parameter was recorded significantly more frequently on the apical view than on the subxiphoid view (Table 2).

The subpopulation of 65 patients with adequate apical and subxiphoid windows were not significantly different from the overall population, except for the proportion of acceptable apical windows (27% versus 21%; *P *= 0.04 for the overall population and subpopulation, respectively). The various parameters obtained on each of these two views were compared in this group of 65 patients (Table 3). No significant difference was observed for the parameters based on the two-dimensional mode: LVEF, RVEDA, LVEDA, and the RVEDA/LVEDA ratio. In the Doppler mode, Ao VTI, E, A, Ea, and E/Ea ratio were significantly decreased on the subxiphoid view, suggesting inadequate alignment between the cursor and blood flow. However, E and A velocities on the subcostal view were underestimated in the same proportion, so the E/A ratio was equivalent to that observed on the apical view.

**Table 3:** Echocardiographic measurements in apical and subxiphoid windows in the subpopulation of 65 patients (except for A and E/A; *n *= 51)

	Apical window	Subxiphoid window	*P *value
**LVEF, %**	54 ± 16	55 ± 14	0.9
**Ao VTI, cm**	18 ± 5	16 ± 5	0.001
**E, cm/sec**	80 ± 25	59 ± 24	0.001
**A, cm/sec**	75 ± 32	55 ± 29	0.001
**E/A**	1.2 ± 0.9	1.1 ± 0.5	0.3
**Ea, cm/sec**	9.5 ± 3.7	8.8 ± 2.8	0.009
**E/Ea**	9.6 ± 4.6	7.6 ± 4	0.001
**RVEDA, cm^2^**	13 ± 6	13 ± 6	0.9
**LVEDA, cm^2^**	26 ± 8	27 ± 8	0.3
**RVEDA/LVEDA**	0.5 ± 0.19	0.49 ± 0.18	0.2

Ao VTI, velocity time integral of aortic blood flow; E/A, ratio between early and late mitral inflow; E/Ea, ratio between early mitral inflow and displacement of the mitral annulus on tissue Doppler imaging; LVEF, left ventricular ejection fraction; RVEDA/LVEDA, ratio between right and left ventricular end-diastolic area.

Similar differences were observed when only patients with a history of COPD or mechanically ventilated patients were considered (data not shown).

**Figure 1:**
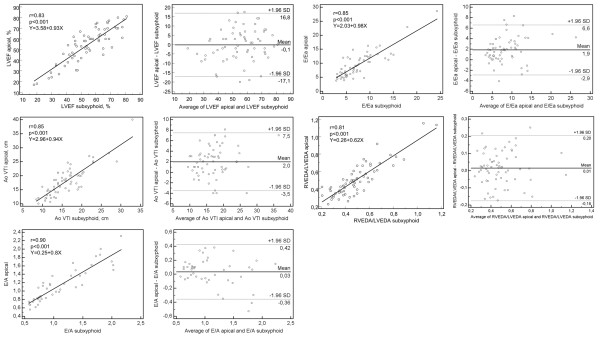
**Regression plot and Bland-Altman analysis between apical and subxiphoid views for the various parameters in the subpopulation of 65 patients**. (LVEF, left ventricular ejection fraction; Ao VTI, velocity time integral of aortic blood flow; E/A, ratio between early and late mitral inflow; E/Ea, ratio between early mitral inflow and displacement of the mitral annulus on tissue Doppler imaging; RVEDA/LVEDA, ratio between right and left ventricular end-diastolic area.

A strong correlation was observed between apical and subxiphoid values for all parameters (Figure 1). The Bland-Altman graphs are presented in Figure 1. The percentage bias, percentage error, and precisions of the apical and subxiphoid views are presented in Table 4 for each parameter. All parameters presented a better precision (lower percentage value) on the subxiphoid view than on the apical view, but two parameters (Ao VTI and E/Ea) presented a bias >10% (12% ± 16 and 22% ± 28, respectively) and therefore did not meet the *a priori *defined criteria.

**Table 4:** Statistical analysis of the various parameters obtained on the subxiphoid view and on the reference apical view

	Mean apical	CV apical	CE apical	Precision of apical	Mean subxiphoid	Bias (± SD)	Bias (% of the apical value)	Percentage error	Precision of subxiphoid
LVEF	54% ± 16	29.6	17.1	34%	55% ± 14	-0.1 ± 8.6	-1.5% ± 17	32%	12%
Ao VTI	18 cm ± 5	29.1	16.8	34%	16 cm ± 5	2.0 ± 2.8	12% ± 16	31%	13%
E/A	1.2 ± 0.9	75.7	43.7	87%	1.1 ± 0.5	0.03 ± 0.2	5% ± 16	33%	81%
E/Ea	9.6 ± 4.6	47.5	27.4	55%	7.6 ± 4	1.9 ± 2.4	22% ± 28	50%	23%
RVEDA/LVEDA	0.5 ± 0.19	37.5	21.6	43%	0.49 ± 0.18	0.01 ± 0.1	3% ± 22	40%	16%

CV, coefficient of variation; CE, coefficient of error.

**Figure 2:**
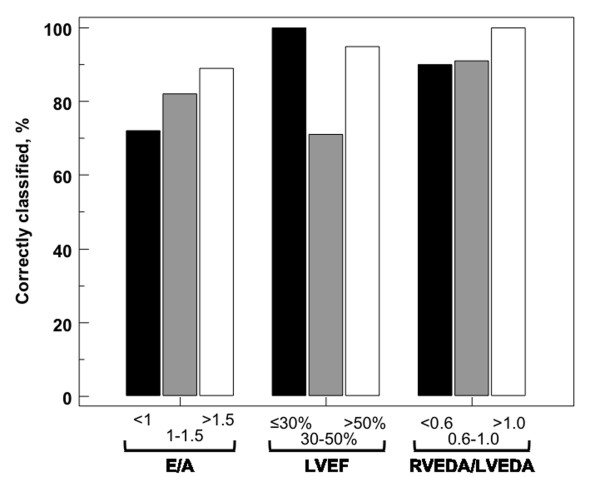
**Accuracy of subxiphoid view for classifying various hemodynamic parameters according to the apical view in the subpopulation of 65 patients**. (LVEF, left ventricular ejection fraction; E/A, ratio between early and late mitral inflow; RVEDA/LVEDA, ratio between right and left ventricular end-diastolic area).

Semiquantitative evaluation of the diagnostic accuracy of the subxiphoid view was determined for the three parameters selected (LVEF, E/A, and RVEDA/LVEDA) by identifying several previously published criteria obtained on the apical view (Figure 2).

## Discussion

In this study, the apical view was the view most frequently obtained (86%), and only a small proportion (5%) of patients did not have an adequate apical view, but had an adequate subxiphoid view. These results emphasize the good feasibility of the apical view in TTE, even in ventilated or COPD patients. This study also shows that the subxiphoid view is a reliable view to study the right- and left-ventricular morphology and function on two-dimensional echocardiography, but not in Doppler mode. The subxiphoid view should be used to explore only the vena cava, pericardium, and the morphology of the left or right ventricles.

The useful window most frequently obtained in our population was the apical window (86%), followed by the parasternal window (66%), and finally the subxiphoid window (49%), as already reported [11-13]. Only 5% of the patients of our population presented an inadequate apical view and an adequate subxiphoid view. Moreover, a greater number of parameters were recorded on the apical view than on the subxiphoid view. Altogether, these results emphasize the better feasibility of the apical view compared with the subxiphoid view in TTE.

The proportion of mechanically ventilated patients in our population was 45%, which is lower than the proportion usually reported in ICU [14]. However, in our study, TTE was performed within the first 2 days after admission of the patients and mostly at the time of admission. We do not believe that a higher proportion of mechanically ventilated patients would have modified our conclusions, as the proportion of adequate views was significantly higher for the apical view than for the subxiphoid view in the population of mechanically ventilated patients (Table 2).

The subxiphoid view is an essential element of echocardiography in an ICU, particularly to analyze the inferior vena cava (for example, size and collapsibility) and pericardium (for example, pericardial effusion). According to our results, only parameters recorded in two-dimensional mode (LVEF and RVEDA/LVEDA) can be recorded on the subxiphoid window without any bias. The bias of E/A was almost nil, but this ratio alone does not constitute a reliable parameter to analyze LV filling pressure in this setting of critically ill patients [15]. The fairly large limits of agreement in the Bland-Altman representations for LVEF, E/A, and RVEDA/LVEDA can also be an argument in favor of the use of a semiquantitative approach for these parameters.

Figure 2 demonstrates the low rate of misclassified patients. Use of the subxiphoid window to analyze ventricular morphology therefore allows a point-of-care approach: preserved or altered LVEF, or normal or dilated right ventricle [16,17].

The discrepancy between apical and subxiphoid values for E, A, E/Ea, and Ao VTI can be explained by the angle between the Doppler axis and blood flow or myocardial movement in the various acoustic windows. With the Doppler mode, red blood cell velocity (or myocardial tissue movement on tissue Doppler) depends on the cosine of the angle between the axis of the Doppler beam and blood flow (myocardial tissue movement): the smaller the angle, the higher the velocity [18]. On the apical view, the heart is examined in the longitudinal (anteroposterior) axis, ensuring perfect alignment between the Doppler beam and blood flow through the mitral or aortic rings and maximum velocities. On the subxiphoid view, the heart is viewed in a different axis (inferoanterior). Consequently, on this view, the alignment between the Doppler beam and aortic flow is not perfect, and the velocity reported by the echocardiographer will be underestimated. Interestingly, the E/A ratio is not modified on the subxiphoid view, as the E and A waves are both decreased to a similar degree. The velocity of the mitral annulus (Ea) is measured in tissue Doppler mode. On the apical view, the Doppler beam is aligned with the longitudinal movement of the mitral annulus, but the subxiphoid view measures the radial movement of the annulus, accounting for the lower Ea velocity obtained in this window. It is noteworthy that the E/Ea and Ao VTI were also underestimated in patients with a history of COPD and in mechanically ventilated patients, two situations theoretically associated with a vertical heart axis that could have resulted in better alignment on the subxiphoid view.

An adequate apical view could not be obtained in the present study in fewer than 15% of cases, whereas use of the subxiphoid view was incomplete, as Doppler analysis appears to be unreliable. Therefore, in the unusual situation of absence of an apical window, the use of another monitoring device should be considered (particularly in intubated patients, in whom transesophageal echocardiography can usually be performed).

This study presents several limitations. The lower velocities obtained on the subxiphoid view are due to the poor alignment between the Doppler axis and blood flow. Some echocardiography devices apply a correction of this angle by aligning the Doppler beam with blood flow, but this angle correction was not tested in this study. However, accurate determination of the appropriate angle correction for velocity is difficult, essentially because this angle can be measured only in a two-dimensional plane, which therefore disregards the angle in the third dimension of the heart. Moreover, angle correction would not be sufficient to correct the velocity of Ea, as the observed decrease is not exclusively due to poor alignment, but also because this view measures a different relaxation property of the myocardium: radial instead of longitudinal. Not all hemodynamic parameters were evaluated by TTE in this study. Right ventricular function was not extensively analyzed, apart from RVEDA/LVEDA (an important parameter of biventricular interaction). However, the misalignment observed in Doppler mode would also be expected to affect measurement of the tricuspid proto-systolic S-wave velocity on tissue Doppler, tricuspid annular plane systolic excursion in M mode (TAPSE), or pulmonary artery pressure. We also did not study the variation of aortic blood flow during passive leg raising or mechanical ventilation to predict fluid responsiveness, as this would have required a strictly selected population [19]. Although the position of the heart and consequently our findings should not differ according to the reason for admission, it would be interesting to confirm our conclusions in a specific population of acute circulatory failure patients.

## Conclusions

Echocardiography comprises a combination of various acoustic windows. The subxiphoid view is already established as an essential part of echocardiography in the ICU to explore the inferior vena cava and pericardium. The results of this study also emphasize that the subxiphoid view cannot replace the apical view to perform complete hemodynamic assessment. In the absence of an apical view, the subxiphoid view can provide only partial information based on analysis of two-dimensional images. To obtain a complete hemodynamic examination, echocardiographers should therefore record both apical and subxiphoid views or use another monitoring device, such as transesophageal echocardiography.

## Key messages

• The apical view appears to be more accurate and more feasible than the subxiphoid window, even in mechanically ventilated or COPD patients.

• The E/Ea ratio and Ao VTI appear to be significantly underestimated on the subxiphoid view and therefore should not be recorded.

• In addition to its ability to explore the vena cava and pericardium, the subxiphoid view can provide information based on morphologic analysis of the ventricles in two-dimensional mode.

## Abbreviations

Ao VTI: velocity time integral of aortic blood flow; BMI: body mass index; CE: coefficient of error; COPD: chronic obstructive pulmonary disease; CV: coefficient of variation; E/A: ratio between early and late mitral inflow on pulsed Doppler; E/Ea: ratio between early mitral inflow and displacement of the mitral annulus on tissue Doppler imaging; HR: heart rate; ICU: intensive care unit; LOA: limit of agreement; LVEDA: left ventricular end-diastolic area; LVEF: left ventricular ejection fraction; MAP: mean arterial pressure; PEEP: positive end-expiratory pressure; PSV: pressure support ventilation; RVEDA: right ventricular end-diastolic area; SAPS2: simplified acute physiologic score 2; TTE: transthoracic echocardiography; VC: volume controlled.

## Competing interests

The authors declare that they have no competing interests.

## Authors' contributions

JM and AS contributed to the acquisition and analysis of data and drafting the manuscript

JM and MS contributed to the conception and design of the study. CT, ZM, GC, and MS were involved in interpretation of data, critically revising the manuscript, and giving final approval. All authors read and approved the final manuscript.
